# Multinational collaboration in solving a European *Salmonella* Braenderup outbreak linked to imported melons, 2021

**DOI:** 10.2807/1560-7917.ES.2024.29.1.2300273

**Published:** 2024-01-04

**Authors:** Hannah L Moore, Martine Aabye, Ann Hoban, Bettina Rosner, Stine K Lefevre, Eva Litrup, Luise Müller, Steen Ethelberg, Sandra Simon, Sooria Balasegaram, Lesley Larkin, Cecilia Jernberg, Johanna Takkinen, EU/EEA/UK S. Braenderup Outbreak Investigation Group, Members of the EU/EEA/UK S. Braenderup Outbreak Investigation Group, Lin Brandal, Derek Brown, Lynda Browning, Marie Anne Chattaway, Ondřej Daniel, Niall deLappe, Rikard Dryselius, Sarah Gee, Nathalie Jourdan Da Silva, Nadja Karamehmedovic, Stine Kjaer Lefévre, Heidi Lange, Wesley Mattheus, Paul McKeown, Joël Mossong, Anaïs Painset, Maria Pardos de la Gandara, Roan Pijnacker, Catherine Ragimbeau, Ruska Rimhanen-Finne, Ife Slegers-Fitz-James, Michaela Špačková, Anni Vainio, Dieter Van Cauteren, An Van den Bossche

**Affiliations:** 1 UK Field Epidemiology Training Program (UK FETP), UK Health Security Agency, London, United Kingdom; 2 UK Health Security Agency, London, United Kingdom; 3 European Programme for Intervention Epidemiology Training (EPIET), European Centre for Disease Prevention and Control, (ECDC), Stockholm, Sweden; 4 Statens Serum Institut, Copenhagen, Denmark; 5 The Danish Health Authority, Copenhagen, Denmark; 6 Robert Koch Institute, Berlin, Germany; 7 Department of Public Health, University of Copenhagen, Copenhagen, Denmark; 8 Robert Koch Institute, Wernigerode, Germany; 9 European Centre for Disease Prevention and Control (ECDC), Stockholm, Sweden; 10 The members of the group are listed under Collaborators

**Keywords:** salmonella, outbreak, EU, food-borne, surveillance, collaboration, policy

## Abstract

A genomic cluster of *Salmonella* Braenderup ST22, a serovar of *Salmonella enterica* subsp. *enterica* which causes symptoms of gastrointestinal illness, was notified by Danish authorities to the European Centre for Disease Prevention and Control (ECDC) on 3 May 2021. By 6 July 2021, *S.* Braenderup outbreak cases (n = 348) had been reported from 12 countries in the European Union/European Economic Area (EU/EEA) and the United Kingdom (UK), including 68 hospitalised cases. With support from affected EU/EEA countries, and in partnership with the European Food Safety Authority (EFSA), ECDC established an international outbreak investigation team to rapidly identify the source and prevent outbreak spread. Consumption information was shared with affected countries through a standard line list, revealing that 124 of 197 cases (63%) reported having eaten (any) melons within 7 days prior to disease onset. The speed and completeness of the investigation, which identified the outbreak vehicle as galia melons imported from Honduras in June 2021, was a direct result of extensive collaboration and information sharing between countries’ national food safety and public health authorities. This article describes the outbreak and the benefits, successes, and challenges of multi-country collaboration for consideration in future large foodborne outbreaks across Europe.

**Key public health message**

**What did you want to address in this study?**

There is no standard approach to outbreak investigations for food and waterborne diseases that are often resource-intensive and involve several countries. We present an example of collaboration across countries, rapid information sharing and harmonised data collection templates with some key lessons learned, which could form the basis of standard practices in multi-country outbreaks.

**What have we learnt from this study?**

Multi-country collaboration can solve outbreaks rapidly, particularly when supported by a supranational organisation. It can help detect outbreaks through sharing of genomic data, conserve resources by distributing the workload between countries and also strengthen conclusions where findings differ compared with individual country investigations.

**What are the implications of your findings for public health?**

Examples of successful practice discussed in the article, for example the use of a standard line-list template and interview questions, could be replicated in other multinational outbreaks to improve speed and completeness of investigations. Examples of challenges, such as different nomenclature of food items, could be considered for the same reason.

## Introduction

Multinational food-borne outbreak investigations require cross-sectoral collaboration not only at the local, regional and national level, but also across countries. This typically involves a number of organisations such as health authorities, regulatory food and veterinary authorities, central and regional laboratories as well as clinicians. Successful outbreak investigation is built on a coordinated approach at all regulatory and administrative levels. This paper presents an example of how a coordinated effort with cross-national collaboration was beneficial in solving a multinational outbreak of *Salmonella* Braenderup in Europe in the spring of 2021.

*S.* Braenderup is a serovar of *Salmonella enterica* subsp. *enterica,* which causes symptoms of gastrointestinal illness including abdominal cramps, diarrhoea, nausea and fever [1]. *S*. Braendrup ranked 19th among *Salmonella* serovars reported to the European Surveillance System (TESSy) between 2015 and 2019 and around 300 cases of *S.* Braenderup in the EU/EEA are reported each year.

## Outbreak detection

On 3 May 2021, a genomic cluster of *S.* Braenderup ST22 was notified by Denmark to the European Centre for Disease Prevention and Control (ECDC) via the Epidemic Intelligence Information System, now called the European surveillance portal for infectious diseases (EpiPulse). On the same day, Belgium reported an increase in *S*. Braenderup cases, including four isolates that had been sequenced and clustered genetically with the Danish outbreak strain. Two days later on 5 May 2021, the United Kingdom (UK) notified an outbreak of a specific single nucleotide polymorphism (SNP) single-linkage cluster of *S.* Braenderup through the European Commission’s Early Warning and Response System (EWRS). By 20 May 2021, more than 200 *S*. Braenderup isolate sequences clustering within 0–6 allelic differences with the Danish outbreak strain had been reported from nine countries (later 12) in the European Union/European Economic Area (EU/EEA), the UK, Canada, Switzerland and the United States (US).

As the reported numbers in May 2021 were markedly higher than was expected at that time of year in Europe [2], a multi-country outbreak was declared and ECDC initiated coordinated outbreak investigations by holding the first international coordination meeting with affected countries on 12 May 2021.

Here we describe the successful investigation and detail the methods leading to effective coordination across affected countries, which resulted in rapid resolution of the outbreak caused by seasonal food.

## Methods

### Coordination of the international outbreak investigation

With support from the affected countries (Austria, Belgium, Czechia, Denmark, Finland, France, Germany, Ireland, Luxembourg, the Netherlands, Norway, Sweden and the UK), ECDC established an international team consisting of members from these countries to investigate the outbreak and identify the source. Under ECDC coordination, affected countries participated in international coordination meetings where epidemiological and laboratory findings, developments in national investigations and working hypotheses were discussed and information shared by each country’s outbreak investigation team. A common case definition was agreed upon and countries shared their raw whole genome sequencing (WGS) data with ECDC for a centralised WGS analysis. This enabled a centralised cluster analysis methodology where a common case definition for a laboratory confirmed case could be verified. The European Centre for Disease Prevention and Control monitored the development of the outbreak in close collaboration with the European Food Safety Authority (EFSA).

### Case definition

A possible outbreak case was defined as a laboratory-confirmed *S.* Braenderup case with symptom onset on or after 15 March 2021 (date of sampling or date of receipt by the reference laboratory if date of onset was not available). A confirmed outbreak case was defined as above and also fulfilling laboratory criteria [2] of a genetically related *S.* Braenderup ST22 isolate according to one of the following: (i) national SNP pipeline within six SNPs; (ii) national core genome (cg) multilocus sequence typing (MLST) pipeline within five cg-allelic differences (AD) with the representative outbreak strain accessible through the European Nuleotide Archive (ENA) code ERR5863130 and in EnteroBase [3] code 2104T8198; (iii) clustering in a centralised WGS analysis within five cg-allelic differences in a single-linkage analysis; (iv) belonging to the same cgMLST HC5_ 259996 cluster (Enterobase scheme); or (v) belonging to a 5-SNP single linkage cluster with SNP designation 1.1.39.57.631.725.% (t5:725) according to the UK Health Security Agency (UKHSA) pipeline.

### Epidemiological investigations

Initial trawling questionnaires from case interviews in the UK and Denmark, and preliminary epidemiological studies in the UK, had suggested fruits, specifically melons (other than watermelons) as a possible vehicle of infection. Subsequently, ‘small melons’ formed a working hypothesis.

A common questionnaire template, modified from the original trawling questionnaires, was developed with a focus on melons and distributed to all affected countries to support their case interviews. Public health authorities performed telephone and online interviews using the template as required but modified as appropriate for their national situation. This was particularly important due to varying terms used for different melon types in different countries. By sharing photographs of different types of melon, the international outbreak investigation team ensured that data on similar types of melons were queried and compiled to a common line list from the country-specific questionnaires. Countries who were able to perform analytical studies due to larger case numbers shared hypotheses and results with all affected countries in order to support other national investigations.

Cases were asked about illness (date of symptom onset; duration; hospitalisation; household members with symptoms of gastrointestinal illness before or after disease onset) and food consumption. Information on food consumption within 7 days prior to illness included places of food consumption outside the home; places where food items had been purchased before disease onset; consumption of a variety of fruit items including various types of melons (Galia, honeydew, cantaloupe and similar melons and watermelon); participation in specific group activities or festivities; travel abroad. Information from questionnaires was collated in a shared line list, with any other notable details provided as comments. This information was then analysed to identify the most common food exposures across all countries to produce a descriptive epidemiological analysis. Cases were described by age, sex and geographical distribution.

Three countries, the UK, Denmark and Germany, performed country-specific case-control studies with the main objective of exploring the hypothesis of melons or other similar types of fruits being the vehicle of infection in the outbreak. Study methodology varied slightly between the three countries and the key differences are described below. Other countries shared sequencing and case interview data to contribute to ongoing investigations.

Controls in the UK and German studies, recruited via a market research panel, were queried about the period of time 7 days before the online questionnaire completion date (UK study), or 7 days before the telephone interview (German study). Controls in the Danish study were randomly selected via the national civil register and asked to report on food consumption 14 days before the telephone interviews. In the UK, questionnaires were completed between 25 May and 26 May 2021, in Denmark, telephone interviews were conducted between 15 May and 20 May 2021 and in Germany, telephone interviews were conducted between 1 July and 27 July 2021. In the German study, to account for a seasonal effect, controls were also interviewed about fruit consumption in the 7 days after Easter Sunday (5–11 April 2021), as this time period was closer to the time period cases were asked about (dates of disease onset of interviewed cases: 31 March–24 May 2021). Three to four controls were matched to each case for all studies. Further details on the recruitment of controls are included in the Supplementary material.

Data were analysed using Excel (Microsoft 2016), R version 4.0 (UK and Denmark) [4] and Stata version 17 (Germany) [5]. Data were analysed using logistic regression and matched logistic regression, as appropriate, to determine adjusted odds ratios (aOR) and 95% confidence intervals (CI). For multivariable analyses, variables with p values < 0.1 from the single-variable analyses were added to the model in a forward stepwise (UK and Denmark) or removed from the model in a backward stepwise (Germany) approach.

To take into account national differences, results from each country were interpreted separately and fed back to international meetings coordinated by ECDC to be discussed in the context of further epidemiological, microbiological and trace-back evidence.

### Trace-back investigation

Trace back investigations were undertaken by the relevant authorities in multiple counties [2]. In the UK, food chain investigations also included gathering evidence to understand the seasonality of supply, storage and shelf life of the melons. Additionally, supermarket loyalty card information was obtained to identify specific retailers and batches of fresh produce purchased by cases.

In Denmark, receipts or credit card transcripts were obtained from four cases and investigated for documented purchase of melons other than watermelons. The Danish Veterinary and Food Administration further investigated where the melons had been imported from.

### Phylogenetic analysis

The outbreak strain was characterised as *S.* Braenderup, ST22, eBURST group (eBG) 24, using previously described genomic methods (UK-SNP address 1.1.39.57.631.725% and Enterobase cgMLST HC5 259996) and a reference strain provided by Danish authorities (see Supplementary Table S1). Clustering methodologies are described elsewhere [6,7]. Each country performed their own genomic sequencing and pipelines to determine whether the reported strains belonged to the outbreak, an approach accepted by ECDC as a valid determination of clustering. During this process, ECDC collected sequences of at least one isolate per national cluster/outbreak for a centralised cgMLST analysis to ensure they fulfilled the case definition. For the ECDC centralised analysis, sequences were analysed using BioNumerics version 7.6.3 (Applied-Maths, Sint-Martens-Latem, Belgium), which included trimming using the default Bionumerics 7.6.3 settings. Assembly-based allele calling was performed using the EnteroBase core genome scheme (BioNumerics), resulting in a cgMLST allelic profile per isolate [8]. Hierarchal clustering analysis was performed using single-linkage clustering [9].

### Food sampling investigations

In the UK, due to preliminary epidemiological evidence, 200 Galia melons were tested by the UKHSA Food, Water and Environment National Reference Laboratory. The melons were obtained from a UK wholesaler and originated from one Honduran grower with 140 melons from one consignment and 60 from another consignment. The EN/ISO 6579 method (International Organization for Standardization, 2017) was used to isolate *Salmonella* spp. using 25g core samples from both ends of the melons which were pooled with a rinse of the entire melon surface. Each melon was tested individually. Further details on testing methodology can be found in the Supplementary material and in the ECDC Rapid Outbreak Assessment [2].

## Results

### Communication and coordination

On 10 May 2021, ECDC sent out the first official communication to the affected EU/EEA countries and the UK via an outbreak notification summary, and the first international coordination meeting for affected countries was organised by ECDC on 12 May. Two follow up coordination meetings were held on 19 May and 15 June 2021. In the meetings, epidemiologists and microbiologists from national public health institutes discussed results from case interviews. In the first meeting on 12 May, preliminary data from the UK led to an initial hypothesis that the outbreak could be caused by some type of imported small melon(s), other than watermelons. The international outbreak was first publicly communicated, with contribution from the affected countries, via the ECDC weekly Communicable Disease Threats Report on 21 May 2021 [10].

**Figure 1:**
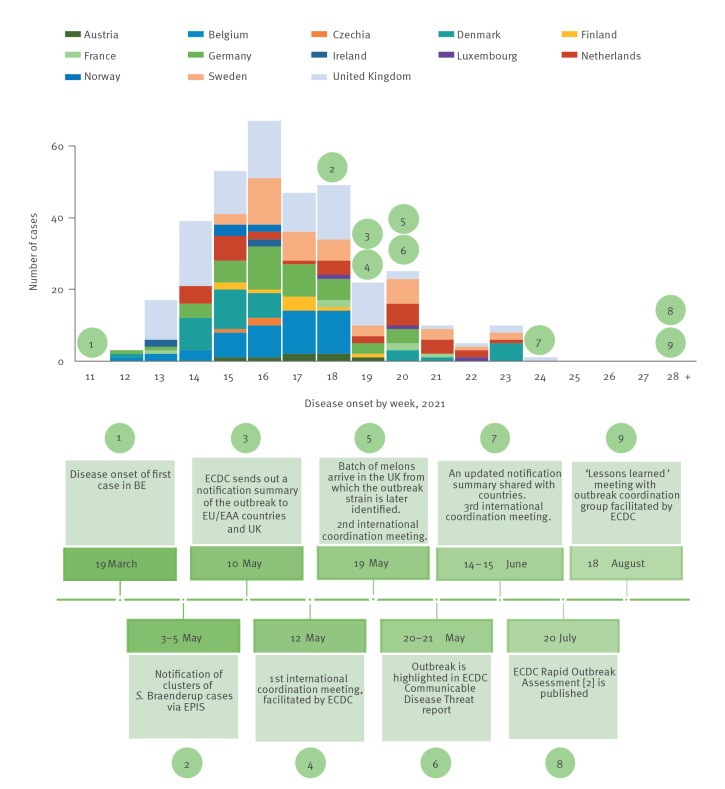
Disease onset by week (or, if unavailable, laboratory receipt or report date) for confirmed *Salmonella* Braenderup ST22 outbreak cases by country and event timeline for coordinating actions, EU/EEA countries (n = 12) and the UK, 19 March–18 August 2021 (n = 348)

BE: Belgium; ECDC: European Centre for Disease Prevention and Control; EPIS: the Epidemic Intelligence Information System (named EpiPulse since 5 July 2021); UK: United Kingdom.

To initiate a standard data collection, ECDC sent out a line list template and the 13 affected countries contributed to the shared line list with case data. As the outbreak continued to evolve, an update of the outbreak notification summary was distributed to affected EU/EEA countries and the UK on 14 June 2021. The European Centre for Disease Prevention and Control shared the first descriptive epidemiology analysis with affected countries on 6 July 2021, as part of the data validation process for the preparation of a joint ECDC-EFSA Rapid Outbreak Assessment which was published on 20 July 2021 [2]. On 18 August 2021, ECDC facilitated the fourth international coordination meeting with affected countries, summing up lessons learned from the outbreak. Figure 1 summarises the timeline of official communications throughout the outbreak.

### Descriptive epidemiology

**Figure 2:**
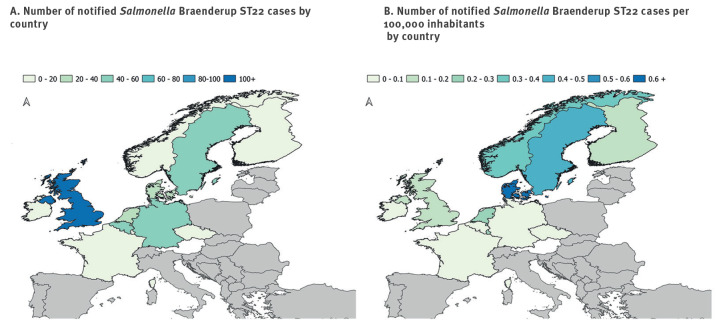
Number of notified *Salmonella* Braenderup ST22 confirmed outbreak cases (n = 348) (A) by country and (B) per 100,000 inhabitants in each country, EU/EEA countries (n = 12) and the UK, 15 March–6 July 2021

By 6 July 2021, 348 confirmed *S.* Braenderup ST22 outbreak cases were reported from 12 countries in the EU/EEA and in the UK, including 68 hospitalisations. In addition, cases were identified in Canada, Switzerland and the US [2]. Figure 2 shows the number of cases in each country and the reported incidence rate per 100,000 inhabitants. Consumption information was available for 197 cases and 124 reported having eaten melons (any type) within 7 days prior to disease onset. Galia melons were the most commonly reported type of melon (46% of the 140 cases for whom information on melons was available).

The number of reported cases and incidence rate in each country is likely affected by the national approach to routine genomic sequencing.

### Analytical epidemiology

The Table shows the results of the three case control studies undertaken by the UK, Denmark and Germany. The UK study showed that compared with controls, for both single variable (data not shown) and multivariable analysis, cases were significantly (p<0.001) more likely to have consumed melons, with an especially strong association with the consumption of Galia melons and cantaloupe melons. Being a case was also positively associated (aOR > 1.00) with the consumption of bananas, tomatoes, fresh or frozen chicken, oranges and leeks in the final model. Using single variable analysis, the Danish study found no association between being a case and the consumption of melons (p = 0.10), but a weak association (aOR = 1.1) was found when using multivariable analysis in a model including ground curry and mangoes. The German study demonstrated by both single variable (data not shown) and multivariable analysis that cases had a significantly higher odds (aOR = 54.7, p = 0.001) of having consumed melons, especially Galia melons, than controls.

**Table:** Overview of multivariable models from case control studies investigating the association between confirmed *Salmonella* Braenderup cases and having consumed food items, United Kingdom, Denmark and Germany, 12–20 May 2021

	Cases exposed		Controls exposed		aOR	95% CI	p value
	n	%	n	%			
United Kingdom Total: 31 cases and 183 controls in the study							
Galia melons	16	52	1	1	671.9	39.0–58,074.0	< 0.001
Cantaloupe melons	9	29	8	4	76.8	5.9–1,761.3	< 0.001
Bananas	29	94	108	59	87.6	5.3–4,564.2	0.01
Tomatoes (type unspecified)	24	77	74	40	9.7	1.6–110.3	0.03
Fresh or frozen chicken	24	77	72	39	6.7	1.4–46.3	0.03
Oranges	18	58	39	21	8.1	1.5–63.0	0.02
Leeks	12	39	8	4	17.2	2.0–222.5	0.02
Denmark Total: 16 cases and 48 controls in the study							
Curry	11	69	19	40	23.6	1.4–403.0	0.03
Mango	5	31	6	13	25.3	1.5–412.0	0.02
Galia melons	3	19	2	4	13.1	1.1–165.0	0.04
Germany Total: 31 cases and 110 controls in the study							
Galia melons	12	38	1	1	54.7	5.7–524.0	0.001
Honeydew melons	12	38	6	5	4.7	1.2–17.9	0.024

aOR: adjusted odds ratio^a^; CI: confidence interval.

^a^ Odds ratio for the German and UK studies were adjusted for sex and age. Odds ratio for the Danish study were matched.

Only food items with a p value < 0.1 in the single-variable analysis were included in the models. For the German study, results shown here are based on reported frequency of fruit consumption by controls in the 7-day period after Easter Sunday (5–11 April 2021).

### Food and environmental sample results

In the UK, *Salmonella* spp. were isolated from two of 200 sampled Galia melons in June 2021. Genomic analysis of the two isolates from melons were confirmed as matching the *S.* Braenderup outbreak strain. Phylogenetic analysis confirmed that one isolate from a melon was the ancestral haplotype and indistinguishable (0 SNPs) from 46 human clinical isolates. The other isolate was two SNPs apart from this, and indistinguishable (0 SNPs) to seven human clinical isolates.

In addition to the Galia melons in the UK, and while not part of active outbreak investigations, the outbreak strain was further identified from a food sample in Austria (pooled peel sample from Galia, cantaloupe and honeydew melons) and from environmental samples in Finland, notably from boot swab samples taken from a hobby henhouse where the chickens had been fed with melon rind. In Germany, the outbreak strain was also identified in a pooled faeces sample from spectacled bears in a zoo. Although melons are part of the normal diet of bears in captivity, it was unclear whether these bears had been fed melons.

### Trace-back investigations

Trace-back analysis from several countries, including the UK and Denmark, and analysis of supply chains pointed towards melons being primarily imported from Honduras. Trace-back and phylogenetic studies are described in detail in the joint ECDC-EFSA Rapid Outbreak Assessment from 20 July 2021 [2].

### Outbreak control measures

Several of the affected countries issued public health and food safety controls and/or public facing advice, e.g. a recall of melons from the suspected producer or recommendations for washing melons before cutting them [2]. Following communication of the epidemiological, phylogenetic and microbiological results from Europe, an investigation was launched by the Honduran authorities. A sample of *S.* Braenderup ST22 matching the outbreak strain was detected on the surface of a washing tank in one of the Honduran facilities where Galia melons are packed and corrective measures were reported to have been taken on-site to prevent future contamination [11]. In December 2021, the European Commission increased the level of official controls on entries of imported Galia melons from Honduras as a result of the outbreak. The frequency of identity and physical checks was set at 10% of consignments entering the EU [12].

## Discussion

A multinational investigation coordinated by ECDC identified melons imported from Honduras as the most likely source of the European outbreak of *S.* Braenderup in the spring of 2021. The conclusion was based on analytical epidemiological studies, trace-back analysis, and the detection of the outbreak strain in samples from melons from Honduras. An investigation in Honduras reported finding the outbreak strain on the surface of a washing tank in one of the Honduran facilities where Galia melons were packed. By July 2021, the outbreak in Europe seemed to have ended, likely due to a shift in the supply chain of melons from Latin America to Southern European countries during the European summer growing season.

The international-level coordination between countries facilitated by ECDC was key in concluding the source of the outbreak in a quick and effective manner. This was particularly important given the absence of confirmatory microbiological evidence of *Salmonella* in melons and the approaching end of seasonal supply of Galia melons from Honduras. Having a supranational coordinating function improved national investigations in terms of information sharing, coordination and consistency in methodology. The international team, communicating via teleconferences, agreed on case definitions, assessed WGS results from different analytical pipelines, aligned case questionnaires (including names of melons) and shared methods for microbiological testing of melons. This ensured much easier interpretation of national findings and allowed effective action to be taken within the context of individual countries. Information sharing was also key in this outbreak. The sharing of WGS results from a country (UK), where routine sequencing of all *Salmonella* isolates is performed, served as an important signal to countries who did not perform routine sequencing to consider sequencing *S.* Braenderup isolates once the outbreak had been detected. Use of a shared line list helped to detect exposure that would have been missed in some countries if the interviews had been undertaken as independent investigations due to small numbers of cases. Sharing results at meetings and in notification summaries at an early stage as well as central coordination with European food authorities meant that efforts were focused on progressing with targeted and prioritised investigations.

Additionally, the international coordination spared resources since it removed the need for full investigations in all affected countries. This was particularly important given the outbreak occurred during the COVID-19 pandemic, where public health and laboratory resources were already under significant strain or diverted to support COVID-19 outbreak response. This is particularly notable for the microbiological testing of melons, given the high number of samples required to detect a positive result and laboratory resource shortages experienced during the pandemic. Conducting coordinated case control studies in parallel in selected countries also spared human resources for other countries and reduced the likelihood of missing a true association. This is illustrated by the weak finding in the Danish study, which may have been disregarded had the German and UK results not been available, which were in agreement and convincing in determining the vehicle of infection. These findings were subsequently confirmed by microbiological and trace-back evidence.

Despite the findings implicating the Galia melons, only half of the cases reported eating melon of any type. One possible explanation is that there was an additional outbreak source (i.e. other melons produced by the same farm), although this seems unlikely considering the evidence. Another plausible explanation could be that cross contamination at various stages in the food chain played a role in this outbreak. For example, during transport/export or during supermarket re-stocking where evidence was obtained in the UK that melon boxes were re-used for other fresh produce, including other types of melons. Recall bias is also a viable explanation. Due to the time span between disease onset and the interview, the exposure period that cases were asked about was farther in the past than the exposure period controls were asked about. Therefore, cases may not have recalled melon consumption as accurately as controls, especially if, for example, melons were consumed outside the home or as one of several ingredients in a composite dish such as a purchased fruit salad. Some interviewees also had difficulty naming the exact type of melons they had consumed and used vague descriptions or terms, highlighting a potential need for the use of images in questionnaires when classifying food items (e.g. Galia vs honeydew vs frog skin melons). Although this is limited in telephone interviews, other methods such as online surveys may be advantageous in these situations. This is particularly important in multi-country investigations, where local terminology can differ and food items may be branded differently.

Both whole and pre-cut melons (which involve additional handling) have previously been reported as sources of outbreaks of *Salmonella*, historically linked to on-farm opportunities for contamination. Ready-to-eat watermelon slices were found to be the source of a high-profile *S.* Newport outbreak affecting six countries in Europe in 2012 [13]. Contaminated cantaloupe melons were identified as the cause of a large *S.* Saintpaul outbreak in Australia, where issues with production and processing methods were implicated [14]. Additionally, melons have been linked to other gastrointestinal illness outbreaks, particularly an outbreak of listeriosis in the US in 2011 (with more than 147 cases in 28 states) [15] and more recently, an outbreak of Shiga toxin-producing *Escherichia coli* (STEC) O157:H7 in the UK [16]. Contamination of melons with non-typhoidal *Salmonella* as well as other pathogens may constitute a notable risk to public health, and it is worth noting for consideration as a potential hypothesis in future outbreaks caused by genetically similar strains *S.* Braenderup and other *Salmonella* spp.

Honduras is one of the main exporters of yellow melons to Europe during the European winter months, with harvest season in Honduras starting around December and usually ending in late spring [2]. *Salmonella* may contaminate melons through a variety of ways, such as from soil where microorganisms are present, from the hands of melon pickers, through irrigation water or during transport and storage. Washing melons immediately post-harvest, a procedure used by the implicated supplier in the outbreak, may also lead to bacterial contamination both externally and internally as melons can absorb contaminated water through the peel, cut stem or any surface cuts or bruises, particularly if the water in the washing tank is not changed for a long period of time. Proper cleaning of the washing tank every time the water is changed could reduce the risk of continued contamination.

Cross-contamination may also occur at home by consumers touching the peel or cutting the melon without washing it first. Micro-organisms can be transferred from the peel to the ingestible flesh of the melon and grow if stored at ambient temperature. There is evidence that *Salmonella* cross-contamination can occur from the affected product to its packaging material, which could explain associations found with illness and other melon types or other fruit if they had been transported together [17]. In this outbreak, contamination at the producer level is most likely considering the widespread distribution of cases over 3 months along with imports of melons and the detection of the outbreak strain on the producer’s packaging site. However, contamination may be transferred between products and producers if common washing tanks or irrigation sources are used. Whether the contamination was solely surface contamination or also occurred within melons could not be determined in this outbreak since samples from peel and interiors were not tested separately. However, this may be of importance in terms of future public health recommendations, i.e. informing consumers about washing melons before cutting them to reduce internal contamination.

## Conclusion

This coordinated collaborative approach across countries and between public health and food safety sectors led to an effective and rapid outbreak investigation of *S.* Braenderup across Europe in the spring of 2021. Galia melons and possibly other small melons from Honduras were identified as the most likely vehicle of infection in this large outbreak. Following lessons from this investigation and other food-borne outbreaks, ECDC and EFSA established the One Health WGS system in June 2022, which will (and already has) notably facilitated cross-border food-borne outbreak investigations. Other experiences from this outbreak could be used as a template or best practice for collaboration in future multi-country outbreaks.

## Supplementary Data

106000 Supplementary Material
