# Supplementary material for: Multinational collaboration in solving a European Salmonella Braenderup outbreak linked to imported melons, 2021
Source: Euro Surveill. 2024 Jan 4;29(1):2300273. doi: 10.2807/1560-7917.ES.2024.29.1.2300273 (PMC10905663; doi:10.2807/1560-7917.ES.2024.29.1.2300273)
Supplement: Supplementary Material [file 23-00273_MOORE_Supplementary_material.pdf]

This supplementary material is hosted by *Eurosurveillance* as supporting information alongside the article "Multinational collaboration in solving a European *Salmonella* Braenderup outbreak linked to imported melons, 2021", on behalf of the authors, who remain responsible for the accuracy and appropriateness of the content. The same standards for ethics, copyright, attributions and permissions as for the article apply. Supplements are not edited by *Eurosurveillance* and the journal is not responsible for the maintenance of any links or email addresses provided therein.

Supplementary material

Methodological information

Recruitment of controls

In all three national case-control studies, cases with a travel history in the 14 days before developing symptoms were excluded.

In the UK study, controls were recruited via a market research panel and frequency matched to cases by age with up to four controls selected per case. Controls were interviewed using an online survey, distributed by a market research panel.

In the DK study, controls matching with cases on age (maximum discrepancy 90 days, though 30 days for children less than 5 years of age), sex and municipality were selected randomly from the Danish Civil Registration. Inclusion of three controls per case was aimed for. To this end, a list of 50 potential control persons were drawn for each case and persons on the list were sought contacted by telephone in consecutive order. If a person refused to participate or did not answer the call, the next person on the list was called.

In the DE study, controls were recruited from the general population by a market research institute on behalf of the Robert Koch Institute (RKI) and frequency-matched to cases on age group, sex, and federal state of residency with three controls selected per case. Controls were interviewed by telephone by the market research institute. For all studies, exclusion criterion for controls was a history of gastrointestinal illness (diarrhoea and/or vomiting) 7 days prior to the interview apart from questions related to the disease, e.g., symptoms.

Microbiological testing

Test methodology used in the UK for melon sampling included using a 25 g sample (cored from the blossom and rind end of each melon) which were pooled and added to a buffered peptone water (BPW) rinse of the melon (prepared by submerging the cored melon for one hour in 225 ml BPW). After overnight incubation of the BPW, secondary enrichment in Rappaport Vassiliadis and Muller-Kauffmann Tetrathionate Novobiocin broths was carried out followed by sub-culture onto Brilliant Green Agar (BGA) and Xylose Lysin Deoxycholate Agar (XLD) (International Organization for Standardization (ISO), 2002). BPW enrichments were also tested by PCR as described

previously [1]. Samples per individual melon were not pooled and 200 melons were tested individually. The melons were obtained from a UK wholesaler and originated from one Honduran grower with 140 from one consignment and 60 from another consignment.

Availability of Genomic data

The table below provides a list of representative sequences for this outbreak that have been made publicly available, and their associated upload databases.

Table S1: Available sequence data linked to outbreak

Database	Uploading country	Project ID	Accession number
ENA	Denmark		ERR5863130*
ENA	Finland	PRJEB21234	SAMEA112144502
ENA	Finland	PRJEB21234	SAMEA112144503
ENA	Finland	PRJEB21234	SAMEA112144504
ENA	Finland	PRJEB21234	SAMEA112144505
ENA	Finland	PRJEB21234	SAMEA112144506
ENA	Finland	PRJEB21234	SAMEA112144507
ENA	Finland	PRJEB21234	SAMEA112144508
ENA	Finland	PRJEB21234	SAMEA112144509
ENA	Finland	PRJEB21234	SAMEA112144510
ENA	Czech Republic	ERP142717	ERR10503085
ENA	Czech Republic	ERP142717	ERR10503084
ENA	Czech Republic	ERP142717	ERR10503083
SRA	England	PRJNA248792	SRR14311522
SRA	England	PRJNA248792	SRR14347855
SRA	England	PRJNA248792	SRR14434536
SRA	England	PRJNA248792	SRR14581133
SRA	England	PRJNA248792	SRR14616023
SRA	England	PRJNA248792	SRR14651714
SRA	England	PRJNA248792	SRR14651730

* denotes reference strain provided by Danish authorities

References

1. Jørgensen F, McLauchlin J, Verlander NQ, Aird H, Balasegaram S, Chattaway MA, Dallman T, Herdman MT, Hoban A, Lai S, Larkin L, McCormick J, Reeves LS, Willis C. Levels and genotypes of *Salmonella* and levels of *Escherichia coli* in frozen ready-to-cook chicken and turkey products in England tested in 2020 in relation to an outbreak of *S.* Enteritidis. International Journal of Food Microbiology [Internet]. 2022 May 16 [cited 2022 Oct 20];369:109609. Available from: https://www.sciencedirect.com/science/article/pii/S0168160522000800
